# Experiences administering the EuroQol five-dimension five-level (EQ-5D-5L) for valuation of health-related quality of life estimates in Kenya: An embedded qualitative study

**DOI:** 10.1371/journal.pgph.0007568

**Published:** 2026-11-04

**Authors:** Dennis Wambiri Waithaka, Angela Kairu, Jacob Kazungu, Stacey Orangi, Felix Masiye, Obinna Onwujekwe, Edwine Barasa

**Affiliations:** 1 Health Economics Research Unit, KEMRI Wellcome Trust Research Programme, Nairobi, Kenya; 2 Department of Economics, School of Humanities and Social Sciences, University of Zambia, Lusaka, Zambia; 3 Health Policy Research Group, Departments of Health Administration & Management and Pharmacology & Therapeutics, College of Medicine, University of Nigeria, Nsukka, Nigeria; 4 Centre for Global Health and Tropical Medicine, Nuffield Department of Medicine, University of Oxford, Oxford, United Kingdom; Ludwig-Maximilians-Universitat Munchen, GERMANY

## Abstract

The EuroQol five-dimension five-level (EQ-5D-5L) instrument is increasingly used to develop health-state utility value sets for computing quality-adjusted life years (QALYs) in economic evaluations. However, little attention has been given to the practical realities of administering EQ-5D-5L valuation interviews in African settings. This study examined experiences of administering EQ-5D-5L valuation interviews in Kenya. We conducted an embedded qualitative study alongside the Kenyan EQ-5D-5L national valuation study. Data were collected through 23 post-valuation in-depth interviews with community participants and 16 debrief sessions with valuation interviewers and field supervisors. Post-valuation interviews were conducted in English or Kiswahili, audio-recorded, transcribed verbatim, translated where necessary, and analysed thematically. Health-state preferences were shaped by personal and contextual factors, including illness experience, household financial implications of ill health, beliefs about death, norms concerning independence and social worth, and local environmental conditions. Participants identified aspects of health they felt were not fully captured by the EQ-5D-5L descriptive system, namely vision, speech, broader aspects of mental health and social relationships. Pain/discomfort, mobility, and usual activities were often the most influential dimensions, while anxiety/ depression was generally given less weight. Tool-related linguistic and semantic challenges affected comprehension of some terms, while participant-related factors such as limited literacy, sensory difficulties and caregiving responsibilities also shaped administration. Valuation interviewers and field supervisors used strategies to preserve the intended logic of the valuation tasks, support participant comprehension, manage interruptions and protect data quality. Embedding qualitative methods within EQ-5D-5L valuation studies can complement standard quality-control procedures and quantitative valuation outputs by explaining how participants understood health states, what factors shaped their preferences, and whether administration challenges may have influenced responses. These insights can help researchers and decision-makers interpret final utility values by showing whether observed valuation patterns reflect locally meaningful preferences or challenges in understanding and completing valuation tasks.

## Introduction

Economic evaluation, which systematically compares the costs and health outcomes of competing interventions, is becoming an increasingly important tool for informing evidence-based resource allocation in low and middle-income countries (LMICs) [1,2]. This growing reliance on economic evaluation also increases the importance of robust and locally relevant approaches to measuring and valuing health outcomes that are used in such evaluations.

Quality-adjusted life years (QALYs) are among the most widely used summary measures of health outcomes in economic evaluations [2–5]. Estimating QALYs requires the use of preference-based instruments that measure individuals’ self-reported health-related quality of life (HRQoL) [2,3]. These instruments may be either disease-specific or generic [3]. While disease-specific measures may capture condition-specific impacts more sensitively, generic instruments are often preferred in economic evaluation because they enable comparisons of health outcomes across multiple disease areas and conditions competing for limited resources [2,3,6].

Among generic HRQoL instruments, the EuroQol five-dimension (EQ-5D) instrument, developed by the EuroQol Group and available in multiple versions, is one of the most widely used globally [1,5,7–11]. The more recent EQ-5D-5L version measures health across five domains (mobility, self-care, usual activities, pain/discomfort, and anxiety/depression) each with five levels of severity ranging from no problems to extreme problems or inability to perform the activity [7]. Responses across these domains are combined to define a health state, which can then be assigned a utility value using a country-specific value set, where 1 represents full health, 0 represents death, and values below 0 represent states considered worse than death [7].

Country-specific value sets are derived from valuation studies conducted with members of the general public in the country for which the value set is being developed [6]. For the EQ-5D-5L, these studies use the EuroQol Group’s standardised EQ-5D-5L valuation protocol [6] and accompanying standard quality-control procedures [12] designed to promote consistency in how valuation interviews are implemented and monitored across countries. The valuation component of these studies is usually administered through structured interviews involving two main tasks: composite time trade-off (cTTO) and discrete choice experiment (DCE). In the cTTO task, participants compare living in a hypothetical health state for a fixed period with living for a shorter period in full health, including considering whether some health states are worse than being dead. In the DCE task, participants choose which of two hypothetical health states they prefer.

Across sub-Saharan Africa, EQ-5D-5L valuation studies are increasingly being conducted to generate contextually relevant utility values, with published value sets now available from countries such as Ethiopia [13], Uganda [14], Ghana [15], and Nigeria [16]. In Kenya, the need for locally derived utility values has become particularly salient as economic evaluation is increasingly being institutionalised as part of efforts to make priority setting and resource allocation more explicit and transparent [17,18]. This institutionalisation is reflected in the establishment of the Benefits Package and Tariffs Advisory Panel in 2025, whose mandate includes reviewing and updating health benefit packages and tariffs using economic evaluation and other health technology assessment considerations [19,20]. The Kenyan EQ-5D-5L national valuation study was therefore timely because it generated locally derived utility values that can strengthen the contextual relevance of economic analyses used in policy and priority-setting decisions [21].

However, the increasing use of EQ-5D instruments in settings beyond those in which they were originally developed and validated raises important methodological and validity questions [5,8,9,11,22]. For instance, socio-cultural and economic differences may influence how participants interpret health-state descriptions and engage with valuation tasks. Moreover, although translation and adaptation of EQ-5D instruments follow a standardised protocol, these processes may not fully capture local meanings and nuances, particularly in multilingual settings and among populations with varying levels of literacy [5,8,9,11,22]. Despite these concerns, comparatively, less attention has been paid to the practical realities of administering EQ-5D instruments in African settings [6,11]. This study, therefore, explored experiences of administering the EQ-5D-5L in Kenya, including how community participants understood and engaged with the EQ-5D-5L domains and valuation tasks; the factors that shaped their health-state preferences; and the administration challenges and coping strategies that emerged during valuation interviews.

## Methods

### Ethics statement

Ethical approval for the embedded qualitative study and the Kenyan EQ-5D-5L national valuation study was obtained from the KEMRI Scientific and Ethics Review Committee (Reference number: KEMRI/SERU/CGMR-C/320/4983). All participants received information about the study and provided written informed consent before participation.

### Study design, setting, and participants

This was an embedded qualitative study because it was integrated within the Kenyan EQ-5D-5L national valuation study [21] to examine how valuation interviews were understood, experienced, and administered in practice. The study was reported in line with the Standards for Reporting Qualitative Research (SRQR) [23], a checklist for reporting qualitative studies, provided as S1 SRQR Checklist. We used SRQR because it provides broader reporting guidance across qualitative study designs and was appropriate for this embedded qualitative study. Fieldwork was conducted in eight counties (Kiambu, Nyeri, Meru, Nakuru, Isiolo, Kakamega, Kisii, and Elgeyo Marakwet) which were purposively selected in the valuation study to reflect socio-economic and cultural diversity in Kenya [21].

Community participants were purposively selected from individuals already enrolled in the valuation study. Selection sought variation in sex, age, EQ-5D-5L language version, and county context (urban, rural, and mixed urban-rural), while also including information-rich or extreme cases identified during valuation interviews, such as participants who declined to trade off any life years in the valuation tasks. Recruitment continued until sufficient variation had been captured and no major new themes were emerging in the interviews, resulting in a final sample of 23 participants, with between two and five participants interviewed per county (Table 1). The resulting sex distribution of the embedded qualitative sample reflected this purposive sampling approach, rather than the sex distribution of the national valuation study sample, which was broadly gender balanced.

**Table 1:** Characteristics of community participants (n = 23).

Characteristic	n (%) or summary
**County**	
Kakamega	3
Elgeyo Marakwet	3
Nakuru	2
Kisii	3
Isiolo	3
Nyeri	2
Kiambu	2
Meru	5
**County context**	
Urban	9 (39.1)
Rural	8 (34.8)
Mixed urban-rural	6 (26.1)
**Sex**	
Male	14 (60.9)
Female	9 (39.1)
**Age (years)**	Median 33; range 19–71
**Age group**	
19–34 years	12 (52.2)
35 years and above	11 (47.8)
**Education attained**	
No formal education	1 (4.3)
Primary	7 (30.4)
Secondary	7 (30.4)
Tertiary (college/university)	8 (34.8)
**Employment status**	
Formal employment	2 (8.7)
Informal employment	12 (52.2)
Not employed	9 (39.1)
**Religion**	
Christian	22 (95.7)
Muslim	1 (4.3)
**Chronic illness**	
Yes	1 (4.3)
No	22 (95.7)
**Tool version**	
Kiswahili	14 (60.9)
English	9 (39.1)

### Data collection

Data were collected between September and October 2025 through post-valuation qualitative in-depth interviews and debrief sessions. We conducted one in-depth interview with each of 23 community participants after they had completed the computer-assisted EQ-5D-5L valuation interview. In addition, 16 debrief sessions were held with the valuation field team, comprising 14 debriefs with valuation interviewers and 2 with field supervisors (DW, AK, and JK).

The valuation interviewer group included 13 individuals selected from 15 undergraduate trainees with health-related backgrounds [21]. Before fieldwork, they completed five days of training covering the EQ-5D and its application, interview content and interviewing skills, field preparation, quality-control procedures and reporting, mock interviews, and supervised trial interviews [21]. Following the training, each valuation interviewer conducted pilot interviews over three weeks and received weekly feedback based on quality-control reports and discussions with the field supervisors [21]. Further details of the valuation interview procedures, interviewer training, and standard quality-control procedures are reported in the main valuation paper [21].

The valuation interview comprised the cTTO and DCE tasks described earlier. During both tasks, participants were encouraged to think aloud by verbalising their thoughts as they worked through the valuation tasks. These verbal reports enabled valuation interviewers to note participants’ reasoning, difficulties, and decision-making processes as they occurred. Similar think-aloud approaches have been used in previous qualitative valuation studies to explore how participants interpret and engage with health-state valuation tasks [4,24].

Post-valuation in-depth interviews were conducted by field supervisors in English or Kiswahili, depending on participant preference, and were audio-recorded using a digital voice recorder. The interview guide explored participants’ understandings of health, their perceptions of the EQ-5D-5L descriptive system and valuation tasks, any aspects of health or wellbeing they felt were not adequately captured, factors shaping their health-state preferences, and their overall experiences of taking part, including any positive or difficult aspects of the process (S1 Text).

In parallel, debrief sessions were held weekly with valuation interviewers and monthly with field supervisors throughout the fieldwork. These sessions were used to reflect on participants’ think-aloud accounts, discuss recurring challenges during administration, identify difficult or unusual cases, and document strategies used to support participant understanding and completion of the tasks. The debriefs also provided a forum for sharing feedback from the standard quality-control procedures in the valuation study.

### Data management and analysis

All interviews and debrief sessions were audio-recorded, transcribed verbatim, and imported into NVivo 12 to support data management and analysis. Interviews conducted in Kiswahili were translated into English after transcription, and the translated transcripts were reviewed for accuracy by the lead qualitative researcher.

Data were analysed using thematic analysis, informed by the study questions and interview guides, while allowing themes to emerge inductively from the data [25,26]. DW first read and re-read an initial set of post-valuation interview and debrief transcripts to become familiar with the data and generate preliminary codes grounded in participants’ accounts. These codes captured, for example, how participants conceptualised health, how they understood and engaged with the EQ-5D-5L tasks, the factors shaping their health-state preferences, and the practical challenges and coping strategies that arose during administration. Coding then proceeded across the full dataset, with related codes grouped and refined into broader themes and sub-themes through an iterative process.

DW led the coding and initial theme development. AK and JK, who alongside DW served as field supervisors, reviewed the emerging codes and themes, and areas of ambiguity or disagreement were discussed until consensus was reached. Where necessary, more complex interpretive issues were discussed with the broader authorship group. Formal inter-rater reliability was not assessed because the analysis was interpretive and thematic rather than designed to quantify coding agreement. Themes were iteratively reviewed, collapsed, expanded, and refined. Illustrative quotes were selected to support the final analysis, with only minor edits made to improve readability without changing meaning.

### Research team and reflexivity

The study team brought expertise in health systems and policy research, health economics, clinical research, qualitative methods, and preference elicitation. Several authors were closely involved in the Kenyan EQ-5D-5L national valuation study, including its design, fieldwork supervision, and interpretation. This created a dual role in which some team members supported implementation of the valuation study while also analysing community participants’ and valuation interviewers’ experiences of that process.

We remained reflexive about how this close involvement may have shaped the analysis. In particular, there was a risk that the qualitative data could be interpreted mainly as operational feedback on fieldwork implementation. To mitigate this, we treated the data both as implementation learning and as evidence of how health was conceptualised, how EQ-5D-5L health states and valuation tasks were understood, and how health-state preferences were formed in practice. Emerging interpretations were discussed across the authorship group, including collaborators who were not directly involved in Kenyan field supervision, to challenge assumptions and maintain attention to both the methodological and contextual meanings of the findings.

## Results

The findings are organised into five interconnected themes that reflect the study’s focus. First, we present findings on the alignment between community participants’ conceptualisations of health and the EQ-5D-5L domains. Second, we explore the factors that shaped participants’ interpretations and valuations of different health states. Third and fourth, we present the main challenges encountered during valuation interview administration, distinguishing between tool- and task-related issues and participant-related issues. Finally, we describe the coping and mitigation strategies used in the field, beyond standard quality-control procedures, to support participant engagement and preserve the quality of valuation data.

### Alignment between community participants conceptualisations of health and EQ-5D-5L domains

Across the counties, most community participants’ descriptions of HRQoL closely reflected one or more of the EQ-5D-5L domains (Table 2).

**Table 2:** Illustrative quotes from participants across counties showing alignment between community participants’ conceptualisations of HRQoL and EQ-5D-5L domains.

Participant ID	Illustrative quote	EQ-5D-5L domain(s) reflected
Post-valuation interview (IDI05_2025_Isiolo)	“Being with complete health is like you can walk, you can bathe yourself, you can do your work without any problems… and not having… depression or… anxiety.”	Mobility; Self-care; Usual activities; Anxiety/Depression
Post-valuation interview (IDI01_2025_ Elgeyo Marakwet)	“Without walking… you cannot do anything… you can do your activities normally and not have that mental or emotional worry… depression.”	Mobility; Usual activities; Anxiety/Depression
Post-valuation interview (IDI15_2025_ Kiambu)	“Good health… is walking, doing your own work, having good thoughts…”	Mobility; Usual activities; Anxiety/Depression (implicitly via “thoughts”)
Post-valuation interview (IDI09_2025 Kakamega)	“To be healthy… you are able to do your usual activities, you are able to dress yourself, you are able to walk…”	Usual activities; Self-care; Mobility
Post-valuation interview (IDI11_2025_Kisii)	“Being healthy is when… you don’t have any pain… you’re not depressed.”	Pain/Discomfort; Anxiety/Depression
Post-valuation interview (IDI22_2025_Meru)	“Being healthy means that you can do your work to 100%… discomforts… mental… or physical.”	Usual activities; Pain/Discomfort; Anxiety/Depression (via “mental discomfort”)
Post-valuation interview (IDI14_2025_ Nakuru)	“To be healthy… is when you can… do your daily activities without any problems and without being assisted…”	Usual activities (and functional independence)

Community participants also demonstrated the ability to compare and prioritise these domains, indicating that they attached different levels of importance to different aspects of health. Across the counties, pain/discomfort, mobility, and doing usual activities, often emerged as the most influential domains shaping participants’ evaluations, whereas anxiety/depression was often treated as less important.

*“When it was pain, walking, and usual activities, and they are extreme or unable to do them they would rather die. But stress and showering, they didn’t have a problem with that.”*

Debrief session (14_2025_Meru)

*“So, they were saying, if I am able to do my usual activities and I don’t have any pain, depression is not an issue.”*

Debrief session (03_2025_Kakamega)

In addition to domains already captured by the EQ-5D-5L, a few participants identified other aspects of health that they felt were not fully reflected in the EQ-5D-5L descriptive system, namely vision, speech, and broader aspects of mental health, and social relationships.

*“When you quarrel with someone… you feel that health is reducing, and that is missing [from the tool]”*

Post-valuation interview (IDI04_2025_Isiolo).

*“One of the community members said that the mental health aspect was not well captured..it is not just depression or anxiety”*

Debrief session (IDI16_2025_Supervisors)

### Factors informing community participants’ health-state preferences

Community participant’s health-state preferences were reportedly shaped by a combination of personal and contextual factors that influenced how they interpreted and traded off different health states across the study counties (Table 3). These factors are summarised in Table 3 and elaborated below, with attention to how each shaped the valuation process.

**Table 3:** Summary of key personal and contextual factors shaping community participants’ health-state preferences across study sites.

Factor	EQ-5D-5L domains or severity levels most affected	Illustrative counties where the pattern was observed
Illness experience: personal or observed	Pain/discomfort; mobility; usual activities; anxiety/depression	Nyeri; Meru; Kisii; Kiambu
Household financial implications of ill health	Severe or extreme limitations in mobility, usual activities, and pain/discomfort.	Kiambu; Meru; Nyeri
Religious and cultural beliefs related to death	Severe or extreme health states across domains	Kakamega; Kiambu; Elgeyo Marakwet
Social norms concerning independence, productivity, and social worth	Usual activities; self-care; mobility	Nyeri; Isiolo; Kisii; Elgeyo Marakwet; Nakuru
Local environmental conditions (terrain and insecurity)	Mobility	Nyeri; Elgeyo Marakwet; Isiolo

Illness experience shaped how participants interpreted and gave salience to specific EQ-5D-5L domains within hypothetical health states. Although only one participant reported having a chronic illness, participants often drew on their own illness experiences or those they had observed among family and community members. When these experiences involved severe difficulties in one or more EQ-5D-5L domains, participants tended to evaluate hypothetical health states containing similar difficulties more negatively, and in some cases traded life years more aggressively, including in response to the wheelchair practice example used in cTTO training.

*“I had a lady who was diagnosed with DVT [Deep Vein Thrombosis] in 2018 and has been on anticoagulants and admitted severally. So, she has experienced extreme pain, unable to do usual activities, not able to walk, so she would rather die many times than experience any of them again”*

Debrief session (DB08_2025_Nyeri)

Household financial implications of ill health shaped preferences by introducing anticipated economic consequences into participants’ valuation of severe health states. This was particularly evident where severe or extreme problems affected mobility, usual activities, or pain/discomfort. Participants reasoned that such states would increase treatment costs, caregiving demands, and loss of income, thereby placing substantial strain on household resources. In some cases, earlier death was framed as a way of protecting the family from further financial burden.

“*Instead of me being in this severe state and probably I’ll still die but I will have consumed a lot of money in treatment… better I die earlier so I don’t go away with all resources, and the other people can be left with peace.”* Debrief session

(DB07_2025_Kiambu)

Religious and cultural beliefs about death shaped preferences by influencing participants’ willingness to engage with the trade-off logic of cTTO. This became most apparent when participants were asked to value severe or extreme health states across domains. For some, life was understood as belonging to God, making it morally inappropriate to choose death even in hypothetical scenarios. In other cases, speaking about death was itself seen as culturally sensitive or taboo, which reduced willingness to trade life years or classify a state as worse than death. In a few instances, these beliefs also led participants to discontinue the interview.

*“Life is not yours, life belongs to God. So, you don’t have the right to take it away.”*

Post-valuation interview (IDI16_2025_Kiambu)

*“In this community (Kakamega), talking about death is like a taboo because it’s like you’re calling it upon yourself.”*

Debrief session (DB15_2025_Supervisors)

Social norms concerning independence, productivity, and social worth shaped how participants interpreted limitations in usual activities, self-care, and mobility. Being unable to perform usual activities or care for oneself was frequently understood not only as a functional limitation, but also as a loss of autonomy, status, and identity. Dependence on others was often viewed as burdensome and undesirable, and in some cases was further associated with stigma and discrimination, contributing to some participants preferring death over health states characterised by severe dependency. This was also evident in responses to the wheelchair example, which some participants regarded as worse than death because it signified severe dependence, social exclusion, and loss of dignity.

*“it is better to die than be on a wheelchair … you are a burden to somebody and this person has to keep pushing you every time...[also] the way they look at you when you are in a wheelchair, you are discriminated”*

Debrief session (05_2025_Nakuru)

*“Here they do not want to be a burden… even my family is going to neglect me… so, they chose dying.”*

Debrief session (DB09_2025_Nyeri)

Local environmental conditions shaped health-state preferences by altering the practical meaning of mobility limitations. In areas with hilly or difficult terrain, inability to walk was seen as practically incompatible with daily life, while in settings affected by insecurity, mobility was linked more directly to personal safety and survival. In these contexts, compromised mobility, including reliance on a wheelchair, was sometimes equated with heightened danger and dependence, contributing to more negative valuations of severe mobility limitations.

*“Because of the terrain… ‘how will I be able to move even with a wheelchair and do anything…I better die.”*

Debrief session (Nyeri DB08_2025)

*“During the raids… the crippled ones… are left back in the community to be killed because you cannot run with them. So, in the tool, they were really prioritizing being able to walk.”*

Debrief session (Isiolo DB11_2025).

### Tool- and task-related challenges

Although the EQ-5D-5L was generally regarded as clear and relevant, some of its features, including the valuation tasks, created challenges during administration. While both valuation tasks were usually completed successfully with appropriate guidance, participants generally found the DCE easier and quicker than the cTTO. By contrast, the cTTO was often experienced as more cognitively and emotionally demanding because it required repeated trade-offs involving years of life, including states considered worse than death.

*“People… didn’t like the TTO… the choosing between A and B in the DCE was actually much easier and faster…”*

Debrief session (Debrief 16_2025_Supervisors).

The kiswahili version of the EQ-5D-5L enabled administration in a language familiar to most participants, but linguistic and semantic challenges (related to wording and transfer of intended meaning) still arose in practice. Some distinctions built into the EQ-5D-5L descriptive system were not easily preserved in everyday spoken Kiswahili. For example, “kidogo” (intended to convey “slight”) and “kiasi” (intended to convey “moderate”) were often understood as interchangeable, reducing the intended gradation between severity levels. Similarly, the formal Swahili expression “kushuka moyo”, used in the EQ-5D-5L to denote depression, was not commonly recognised in everyday speech and was sometimes interpreted literally as a heart problem, or more broadly as discouragement or loss of hope. In a few cases, small differences in wording or pronunciation also altered meaning. For instance, some participants struggled to distinguish “huna” (“you do not have”) from “hakuna” (“there is no”), while others heard “huna” as “una” (“you have”), effectively reversing the meaning of the statement.

*“Even if we speak Kiswahili, many of us do not understand the formal Swahili. For example, when he said ‘kushuka moyo’ I struggle(d)..”*

Post-valuation interview (IDI04_2025_Isiolo)

*“Using ‘kiasi’ and ‘kidogo’ was confusing because when speaking we use them the same way”* Post-valuation interview (IDI05_2025_Isiolo).

*“The word huna, she kept saying it should be hakuna.”*

Debrief session (DB_12_2025_Meru)

Technology-related problems also complicated administration in a small number of valuation interviews. These included laptops freezing mid-interview and occasional missing files, which interrupted the flow of the valuation interview and, in some cases, required parts of the interview to be repeated. Such interruptions raised concerns about altered response patterns on repetition, as participants might recall earlier answers and move through repeated sections more quickly than they had initially.

*“So yesterday one of the computers was hanging quite a lot at the middle of the interview, so [name of valuation interviewer] had to keep on restarting [the laptop] and when you start you start [administering the valuation interview] afresh because the respondent remembers what was happening you might find some interviews sort of hastened than expected and that may be flagged as a quality issue”*

*Debrief session (DB16_2025_Supervisors 2).*

### Participant-related challenges

Participant-related factors also shaped the valuation interview administration. Literacy and formal education influenced how easily some participants engaged with the language and logic of the valuation tasks. In general, participants with higher levels of formal education were described as navigating the valuation tasks more easily, whereas those with less formal education often required more time to grasp the tasks. At the same time, valuation interviewers noted that limited literacy did not necessarily imply limited understanding, as some participants with little formal education were still able to engage well with the tasks.

*“…people who have higher levels of education have little challenges with language but people with lower levels of education… you have to… be explaining over and over again…”*

Debrief session (DB_03_2025_Kakamega)

*“Literacy levels and understanding, they do not go hand in hand here. You can be illiterate here, but the understanding is good.”*

Debrief session (DB09_2025_Nyeri)

Hearing and visual difficulties, particularly among older participants, as well as competing caregiving responsibilities, affected administration by prolonging the valuation interviews, and potentially adding to fatigue or practical burden for both participants and valuation interviewers.

“He couldn’t hear well, so we had to shout… wheelchair example alone took us 20–25 minutes.” Debrief session (DB 15_2025_Meru)

“Problem of visualizing things… you have to zoom…it was taking too much time.” Debrief session (DB 14_2025_Meru)

“It takes a lot of time to interview participants with children… such interviews also tend to take longer.”

Debrief session (DB04_2025_Kisii)

### Coping and mitigation strategies

In addition to the standard quality-control procedures described in the valuation study [21], supervisors and valuation interviewers used regular debrief sessions to identify emerging implementation challenges and agree on practical responses during fieldwork (Table 4). The narrative following Table 4 is organised around four broad purposes of these strategies: preserving the intended logic of the valuation tasks, supporting participant comprehension, managing pace and interruptions, and protecting data integrity where meaningful completion of the valuation interview was at risk.

**Table 4:** Key implementation challenges, strategies applied during valuation interviews, and intended purpose of each strategy.

Key challenge addressed	Strategy applied during valuation interviews	Purpose of strategy
Wheelchair example valued as worse than death during cTTO training	Emphasizing the hypothetical nature of the scenario and, where needed, using a more clearly acceptable better-than-death example	Preserving the intended logic of the valuation tasks
Cultural or religious discomfort with discussing death	Reassuring participants that research participation was voluntary and the scenarios were hypothetical and not predicting or wishing death	
Linguistic and semantic challenges	Clarifying terms using locally familiar expressions while maintaining conceptual meaning	Supporting participant comprehension
Limited literacy or formal education	Reading questions aloud, allowing more time, and providing additional explanation during practice tasks	
Visual limitations when viewing laptop screens	Enlarging text and adjusting screen visibility	
Participants moving too quickly through valuation tasks	Encouraging reading aloud, asking participants to slow down, and probing the rationale for inconsistent responses	Managing pace and interruptions
Divided attention or temporary distractions	Pausing and resuming interviews when participants were ready	
Participants unable to understand the task despite additional support	Discontinuing interviews and replacing participants	Protecting data integrity
Technology-related problems	Restarting laptops and repeating affected sections where necessary	

First, to preserve the intended logic of the valuation tasks, valuation interviewers emphasised the hypothetical nature of the scenarios and used alternative examples where needed. This was especially important when participants interpreted scenarios in ways that could disrupt the standardised valuation protocol. For example, some valued the wheelchair example as worse than death even though it was intended to introduce a better-than-death state, while others expressed cultural or religious sensitivities with death-related trade-offs. In these situations, valuation interviewers clarified the hypothetical nature of the task, reassured participants that the scenarios were not predicting or wishing death, and, where necessary, used more acceptable examples to distinguish better-than-death from worse-than-death states before proceeding.

*“She chose death [during] the wheelchair example... So, I used [other] examples to help her understand the better than death, for example, having a fever [as better than death] versus losing [both] legs or burns [as worse than death].”*

Debrief session (Debrief 12_2025_Meru)

*“He was not willing to give up any life… saying life matters belong to God… we had to explain that it is research and not [based on] a real-life situation.”*Debrief session (Kakamega DB_03)

Second, to support participant comprehension, valuation interviewers used locally familiar phrasing, reading aloud, and additional time during practice tasks. This included adapting formal Kiswahili expressions to terms more commonly used in the local setting and repeating key sections where needed until participants could understand the valuation logic. These strategies helped participants engage with the tasks while seeking to preserve the intended meaning of the health states and valuation constructs. However, they also relied on careful valuation interviewer judgement, particularly in deciding how far to explain or adapt wording without changing the intended meaning of the protocol.

*“I had to change the formal Swahili term ‘kiti cha magurudumu’ for wheelchair to the term used locally ‘kagari kakusukumwa’… otherwise participants in this area found it confusing.”*Debrief session (DB11_2025_Isiolo)

*“Some with very low levels of education took longer… but with explanation and the protocol guiding the survey, they could catch up as we proceeded.”*Debrief session (DB05_2025_Nakuru)

Third, to manage pace and interruptions, valuation interviewers paused interviews where participants were distracted or needed to attend to caregiving responsibilities. Interviews were resumed once participants had attended to these responsibilities and were ready to continue. Where participants moved too quickly, valuation interviewers encouraged them to read health states aloud, slow down, and explain their reasoning. These strategies helped sustain engagement and encouraged more careful consideration of trade-offs. However, they could also lengthen interviews and increase the practical burden on both participants and valuation interviewers.

*“[For] participants with children, sometimes [we] had to stop the interview to attend to the child first… such interviews tended to take longer.”*

Debrief session (DB04_2025_Kisii)

*“He was reading fast… finishing and clicking quickly. I had to tell him to slow down and read everything carefully.”*Debrief session (DB14_2025_Meru)

Finally, to protect data integrity, valuation interviews were discontinued where participants could not meaningfully complete the tasks despite additional support, while practical adjustments were made for visual and technology-related barriers. In some cases, participants who could not understand the task after repeated support during the practice session were replaced. In other cases, valuation interviewers enlarged text to address visual limitations or restarted laptops and repeated affected sections when technology-related disruptions occurred. These responses helped preserve response validity, but also illustrate the limits of standardised implementation in real-world field conditions.

*“If they [illiterate] understand the practice session, then we proceed, but if not, then we stopped. I think we stopped for like 2, but the ones who’ve understood we’ve just done the interview with them.”*

Debrief session (DB16_2025_Supervisors)

## Discussion

This study examined experiences of administering the EQ-5D-5L in Kenya within the context of the Kenyan EQ-5D-5L national valuation study. The findings suggest that EQ-5D-5L valuation in this setting was both technically standardised and socially situated. The embedded qualitative study was therefore important because it allowed us to examine how the standardised valuation protocol was understood and applied in practice. The participants’ engagement with the EQ-5D-5L valuation tasks was shaped by how they understood the health domains, the meanings they attached to different health states, and the practical support provided by valuation interviewers during administration. The discussion below considers what the findings imply for the conceptual fit of the EQ-5D-5L, interpretation of the resulting utility values, and the design and implementation of future valuation studies in similar settings.

The close alignment between participants’ descriptions of health and the domains captured by the EQ-5D-5L suggests that the instrument reflects several core aspects of health that are meaningful and relevant in this setting. This corresponds to what Herdman, Fox-Rushby [10] describe as ‘conceptual equivalence’, that is, whether the underlying concepts measured by an instrument make sense in the local context. In this regard, our findings support the broad suitability of the EQ-5D-5L for national valuation work in Kenya. However, this support for conceptual equivalence was partial rather than complete. While participants recognised the relevance of the EQ-5D-5L domains, they also highlighted aspects of health outside the descriptive system, namely vision, speech, broader mental health, and social relationships. This suggests that recognising the relevance of the core domains does not necessarily mean that the EQ-5D-5L descriptive system fully captures locally meaningful understandings of health. This aligns with wider literature showing that the EQ‑5D captures core aspects of health but may omit certain dimensions that matter in particular settings or populations [27,28]. However, adding extra dimensions does not always improve how well the EQ-5D performs, and each new dimension may increase the time and effort required by valuation interviewers and participants [28]. Further assessment is therefore needed to determine whether locally identified additional aspects of health would meaningfully improve the EQ-5D-5L’s ability to capture health in Kenya, while balancing added local relevance against participant burden, valuation complexity, and comparability across settings.

A clear pattern also emerged in how different health dimensions were prioritised across counties. Pain/discomfort, mobility, and usual activities were consistently described as having the strongest influence, while anxiety/depression was viewed as less central. This pattern aligns with the Kenyan valuation study, where pain/discomfort had the greatest impact on perceived quality of life, followed by mobility limitations, difficulties with usual activities, anxiety/depression, and self‑care [21]. Similar trends have also been reported in Uganda [14] and Ghana [1], where pain/discomfort and mobility carried the greatest influence, whereas anxiety/ depression was placed lower. In contrast, evidence from Ethiopia shows that anxiety/depression exerted the strongest influence on people’s assessment of health [13]. In this study, the lower salience of anxiety/depression appeared to reflect both participants’ prioritisation of more visible or functionally limiting dimensions, such as pain/discomfort, mobility, and usual activities, and linguistic and semantic ambiguity around the anxiety/depression domain. The Kiswahili term “kushuka moyo” was not always recognised as depression and was sometimes interpreted literally as a heart problem, discouragement, or loss of hope, suggesting that lower weighting may partly reflect how this domain was translated and understood rather than mental health being unimportant. Taken together, these findings highlight that, even where the same broad health domains are recognised across settings, the relative importance attached to them may vary in contextually meaningful ways.

The personal and contextual factors that shaped participants’ health-state preferences have important implications for how the resulting utility values should be interpreted. Rather than being treated as sources of bias within the Kenyan valuation study, these factors can be understood as part of how members of the public form preferences about health states within their own social context. This is consistent with qualitative work from the United Kingdom showing that people often make hypothetical health states more concrete by drawing on personal experience and social circumstances [4]. As a result, the Kenyan EQ-5D-5L value set reflects how health states are understood and valued locally. The qualitative findings did not lead to additional post-hoc exclusion of completed valuation data from the final value set; instead, they provided contextual evidence for interpreting the final utility values. However, this also raises important questions about the transferability of value sets across countries. Where contextual drivers of valuation differ substantially between settings, applying value sets from other countries may introduce bias into cost-utility analyses by using utility values that do not adequately reflect local preferences.

At the same time, while our data show the factors participants drew on when reasoning through the valuation tasks, they provide less insight into the decision rules used to arrive at final valuations. For example, it remains unclear whether participants weighed multiple dimensions and severity levels against one another, as assumed in compensatory decision-making, or whether they relied on simpler cognitive shortcuts, such as focusing primarily on the health dimension they considered most important when making a choice [4,29]. This remains an important gap in understanding how participants translate their interpretations of health states into final valuations.

Linguistic and semantic challenges affected administration of the EQ-5D-5L in important ways. Similar issues have been reported in EQ-5D translation and valuation studies in Africa, including translation into Shona in Zimbabwe [22], Xhosa in South Africa [8], and Arabic in Egypt [5]. This suggests that translation should not be viewed as a one-off technical exercise, but as an ongoing process of negotiating meaning, including during fieldwork, particularly in multilingual settings where formal wording, colloquial usage, and oral delivery may not always align.

Participant-related challenges further show that comprehension of valuation tasks is shaped not only by translated wording, but also by literacy, sensory limitations, attention, and the practical circumstances in which interviews are conducted. Similar concerns have been echoed in other valuation studies [5,11]. In Egypt, Al Shabasy, Abbassi [5] found that the standardised valuation protocol was not always well suited to participants with limited literacy or formal education and introduced visual aids (i.e., pictorial representations of the health states and coloured cards using traffic-light system for severity levels), as well as reading health states aloud twice to support comprehension. Likewise in Morocco [30] and Pakistan [11], graphical presentations were reportedly used successfully to facilitate the valuation interviews among participants with limited literacy. Together, these participant-related and linguistic challenges show that even when a standardised valuation protocol is used, participants may still require support that responds to their language, literacy, sensory needs, and interview circumstances.

The coping and mitigation strategies used during fieldwork suggest that making EQ-5D-5L valuation feasible and acceptable in community settings may require structured flexibility during administration. Structured flexibility refers to carefully guided adaptation that responds to field-level challenges while preserving the intended logic of the valuation tasks. In this study, such flexibility enabled valuation interviewers and supervisors to respond to tool-related and participant-related challenges. Similar local adaptations have been reported in other settings. In Egypt [5], the wheelchair training example was replaced with a migraine example after the pilot study showed that many participants regarded it as worse than death. In Vietnam, a third-person approach in cTTO was used because direct discussion of death was considered culturally sensitive [31].

### Study limitations

This study has two main limitations. First, the post-valuation interviews were conducted after participants had completed the EQ-5D-5L valuation interview, which may have influenced how they subsequently described health and reflected on the valuation tasks. In particular, prior exposure to the EQ-5D-5L may have primed participants towards its health domains and response formats. To limit this, the interviews began with open-ended questions about health and wellbeing before participants were asked about the EQ-5D-5L specifically, although this could not fully remove the possibility of priming. This limitation points to the value of staged qualitative designs that collect data before, during, and after valuation tasks: before valuation to capture broader conceptualisations of health prior to exposure to the EQ-5D-5L, during valuation to examine real-time reasoning, and after valuation to explore participants’ experiences of the process. In addition, although participants were encouraged to think aloud during the valuation tasks, such verbal reports may not fully capture all aspects of the reasoning process, including more implicit or difficult-to-articulate influences on choice [4,24,32].

Second, the valuation interviewer and supervisor debriefs were retrospective and may have been influenced by recall bias, selective attention, or post hoc reflection rather than capturing participants’ views directly. In addition, our qualitative data did not allow us to examine in depth other valuation interviewer-related influences on data quality, such as differences in communication style or interviewer–participant interaction [6].

## Conclusion

Administering the EQ-5D-5L was not simply a technical exercise of following a standardised valuation protocol; it was also shaped by how participants understood the health states and valuation tasks, the lived experiences and local realities they brought to them, and the support provided by valuation interviewers. By embedding qualitative methods within the valuation study, we were able to generate insights that would not be apparent from standard quality-control procedures and valuation outputs alone.

These findings have three implications for future EQ-5D-5L valuation studies, particularly in LMICs. First, countries should be supported to generate their own value sets where feasible, because contextual factors can shape how health states are interpreted and valued. Where countries use value sets from elsewhere, they should explicitly consider whether those values are transferable to their own contexts. Second, qualitative methods should be embedded within EQ-5D-5L valuation studies so that final utility values can be interpreted alongside evidence on how participants understood the health states, what contextual considerations shaped their preferences, and whether administration challenges may have influenced responses. This can help clarify whether observed valuation patterns reflect meaningful local preferences or whether they may have been influenced by difficulties in understanding or completing the valuation tasks. Third, valuation protocols and interviewer training should build in structured flexibility, including guidance on how to manage literacy-related needs, linguistic challenges, culturally sensitive concepts such as death, and locally problematic examples while preserving the intended logic of the valuation tasks. These steps could strengthen the local relevance, feasibility, credibility, and interpretation of EQ-5D-5L valuation studies in diverse settings.

## Supporting information

S1 SRQR Checklist Standards for Reporting Qualitative Research checklist. (DOCX)

S1 Text Data collection guides. (DOCX)
