# Supplementary material for: Experiences administering the EuroQol five-dimension five-level (EQ-5D-5L) for valuation of health-related quality of life estimates in Kenya: An embedded qualitative study
Source: PLOS Glob Public Health. 2026 Nov 4;6(11):e0007568. doi: 10.1371/journal.pgph.0007568 (PMC13734439; doi:10.1371/journal.pgph.0007568)
Supplement: Supplementary file 1 [file pgph.0007568.s001.docx]

**Experiences administering the EQ-5D-5L for valuation of health-related quality of life estimates in Kenya: An embedded qualitative study: SRQR Checklist**

| **Topic** | **No.** | **Item** | **Page No.** |
| --- | --- | --- | --- |
| Title/Abstract | S1 | Concise description of the nature and topic of the study. Identifying the study as qualitative or indicating the approach (e.g., ethnography, grounded theory) or data collection methods (e.g., interview, focus group) is recommended. | Pg. 1, Lines 1-3 |
|  | S2 | Summary of key elements of the study using the abstract format of the intended publication; typically includes background, purpose, methods, results, and conclusions. | Pg. 2, Lines 18-50 |
| Introduction | S3 | Description and significance of the problem/phenomenon studied; review of relevant theory and empirical work; problem statement. | Pg. 3-5, Lines 52-98 |
|  | S4 | Purpose of the study and specific objectives or questions. | Pg. 5, Lines 92-98 |
| Methods | S5 | Qualitative approach (e.g., ethnography, grounded theory, case study, phenomenology, narrative research) and guiding theory if appropriate; identifying the research paradigm (e.g., postpositivist, constructivist/interpretivist) is also recommended; rationale. | Pg. 6, Lines 101-102; |
|  | S6 | Researchers' characteristics that may influence the research, including personal attributes, qualifications/experience, relationship with participants, assumptions, and/or presuppositions; potential or actual interaction between researchers' characteristics and the research questions, approach, methods, results, and/or transferability. | Pg. 10-11, Lines 171-194 |
|  | S7 | Setting/site and salient contextual factors; rationale. | Pg. 6, Lines 104-106 |
|  | S8 | How and why research participants, documents, or events were selected; criteria for deciding when no further sampling was necessary (e.g., sampling saturation); rationale. | Pg. 6, Lines 107-113 |
|  | S9 | Documentation of approval by an appropriate ethics review board and participant consent, or explanation for lack thereof; other confidentiality and data security issues. | Pg. 11, Lines 195-199 |
|  | S10 | Types of data collected; details of data collection procedures including (as appropriate) start and stop dates of data collection and analysis, iterative process, triangulation of sources/methods, and modification of procedures in response to evolving study findings; rationale. | Pg. 8-9, Lines 120-150 |
|  | S11 | Description of instruments (e.g., interview guides, questionnaires) and devices (e.g., audio recorders) used for data collection; if/how the instrument(s) changed over the course of the study. | Pg. 8-9, Lines 138-150 |
|  | S12 | Number and relevant characteristics of participants, documents, or events included in the study; level of participation (could be reported in results). | Pg. 6-9, Lines 118-150; Table 1 |
|  | S13 | Methods for processing data prior to and during analysis, including transcription, data entry, data management and security, verification of data integrity, data coding, and anonymization/deidentification of excerpts. | Pg. 9, Lines 152-155 |
|  | S14 | Process by which inferences, themes, etc., were identified and developed, including the researchers involved in data analysis; usually references a specific paradigm or approach; rationale. | Pg. 9-10, Lines 156-170 |
|  | S15 | Techniques to enhance trustworthiness and credibility of data analysis (e.g., member checking, audit trail, triangulation); rationale. | Pg. 10-11, Lines 167-194 |
| Results | S16 | Main findings (e.g., interpretations, inferences, and themes); might include development of a theory or model, or integration with prior research or theory. | Pg. 11-25, Lines 200-440 |
|  | S17 | Evidence (e.g., quotes, field notes, text excerpts, photographs) to substantiate analytic findings. | Pg. 11-25, Lines 200-440 |
| Discussion | S18 | Short summary of main findings; explanation of how findings and conclusions connect to, support, elaborate on, or challenge conclusions of earlier scholarship; discussion of scope of application/generalizability; identification of unique contribution(s) to scholarship in a discipline or field. | Pg. 25-29, Lines 442-531 |
|  | S19 | Trustworthiness and limitations of findings. | Pg. 30, Lines 532-550 |
| Other | S20 | Potential sources of influence or perceived influence on study conduct and conclusions; how these were managed. | Pg. 30, Lines 532-550 |
|  | S21 | Sources of funding and other support; role of funders in data collection, interpretation, and reporting. | Pg. 32, Lines 573-576 |

**Reference**

1. O'Brien BC, Harris IB, Beckman TJ, Reed DA, Cook DA. Standards for reporting qualitative research: a synthesis of recommendations. Academic Medicine. 2014;89(9):1245-1251.
