# Supplementary material for: Experiences administering the EuroQol five-dimension five-level (EQ-5D-5L) for valuation of health-related quality of life estimates in Kenya: An embedded qualitative study
Source: PLOS Glob Public Health. 2026 Nov 4;6(11):e0007568. doi: 10.1371/journal.pgph.0007568 (PMC13734439; doi:10.1371/journal.pgph.0007568)
Supplement: Supplementary file 2 [file pgph.0007568.s002.docx]

# **S2 Data collection guides**

*These guides were used flexibly to support data collection with community members, interviewers, and supervisors involved in the EQ-5D-5L national valuation study.*

## **Community interview guide**

*Interviewer note: Use this guide flexibly. Not all probes need to be asked in every interview. Where possible, prioritise participants whose valuation responses appeared unusual, extreme, or especially information-rich during the main EQ-5D-5L interview.*

1. In your view, what does it mean for a person to be healthy in your community?

- What kinds of things make people feel healthy or unhealthy in daily life?
- Are there particular physical, emotional, social, or environmental factors that matter most?

2. Thinking about the questions you answered, to what extent did the tool capture the things that matter for health in your community?

- Did the five areas covered in the tool - mobility, self-care, usual activities, pain/discomfort, and anxiety/depression - reflect your understanding of health? Why or why not?
- Were there any important aspects of health or wellbeing that you felt were missing? If yes, which ones?
- Were some areas covered by the tool more important than others? Why?

3. How did you find the tasks where you were asked to compare living in different health situations or living for different lengths of time?

- Were any parts of these tasks difficult to understand or answer?
- Was one task easier than the other? Why?
- Were there any words, phrases, or ideas that were confusing?

4. When making your choices, what mainly influenced your decisions?

- Did personal experience with illness, disability, caregiving, or treatment influence your thinking?
- Did family roles, religion, culture, or community expectations shape how you viewed different health states?
- Did local conditions, such as terrain, safety, livelihood, housing, or access to services, affect how you judged some health problems?
- Were there some health problems you felt were especially serious? Why?

5. Overall, how was your experience of taking part in the interview?

- What aspects were easy or positive?
- What aspects were difficult or uncomfortable?
- Did you feel the interviewer explained the tasks clearly and respectfully?
- Is there anything you think should be improved if this tool is used again in communities like yours?

## **Interviewer and supervisor debrief guide**

*Facilitator note: Use this guide in debrief sessions with interviewers and supervisors. Focus on recurring patterns across interviews, including unusual or extreme responses, implementation challenges, and strategies used to support participant understanding.*

1. How was your overall experience administering or supervising the EQ-5D-5L valuation interview this week?

- What worked well?
- What challenges arose?
- Were there notable differences across counties or participant groups?

2. How did participants generally understand and engage with the EQ-5D-5L descriptive system and valuation tasks?

- Which questions, concepts, or tasks were easiest for participants to engage with?
- Which were most difficult to explain or answer, and why?
- Were there recurring issues related to wording, translation, or interpretation?

3. What appeared to shape participants’ choices and health-state preferences during the think-aloud process?

- Did participants draw on personal experience, family roles, religion, culture, or community norms?
- Did local contextual factors such as geography, insecurity, poverty, or access to care appear to influence responses?
- Were there unusual or extreme responses, such as refusing to trade life years or assigning very low values? What seemed to explain these responses?

4. How did participants generally respond to sensitive topics, such as death, severe disability, pain, or mental health?

- Were there signs of discomfort, hesitation, or distress?
- How were these situations handled?

5. What strategies did interviewers use to support participant understanding and completion of the tasks?
