# Supplementary material for: Experiences administering the EuroQol five-dimension five-level (EQ-5D-5L) for valuation of health-related quality of life estimates in Kenya: An embedded qualitative study
Source: PLOS Glob Public Health. 2026 Nov 4;6(11):e0007568. doi: 10.1371/journal.pgph.0007568 (PMC13734439; doi:10.1371/journal.pgph.0007568)
Supplement: Supplementary file 3 [file pgph.0007568.s004.docx]

**Response to Reviewers**

| **Manuscript title** | Experiences administering the EQ-5D-5L for valuation of health-related quality of life estimates in Kenya: An embedded qualitative study |
| --- | --- |
| **Journal** | PLOS Global Public Health |
| **Manuscript number** | PGPH-D-26-02009 |

Dear Dr. Noorali and Reviewers,

Thank you for considering our manuscript and for the constructive editorial and reviewer comments. We are grateful that both reviewers found the manuscript technically sound. We have prepared this point-by-point response to show how we have addressed each journal requirement and reviewer comment in the revised manuscript.

**Table 1: Journal requirements**

| **No.** | **Comment** | **Draft response/ action** |
| --- | --- | --- |
| 1. | Please amend the detailed Financial Disclosure statement. It should be in full sentences, clarify all funding sources, grants, grant numbers and organizations, state the initials of each author who received each grant, state the role of funders, and state whether any authors received salary from the funders. | Thank you for this comment. We revised the funding statement to clarify that the study was supported by the Wellcome Trust through grant number 228187 and by the EuroQol Research Foundation through grant number 2219-PCG. We also added that the Wellcome Trust grant supported salaries for EB, SO, FM, and OO. In addition, we clarified that the funders had no role in the study design, data collection and analysis, decision to publish, or preparation of the manuscript, and that the views expressed do not necessarily reflect those of the EuroQol Research Foundation. |
| 2. | Please address restrictions to data sharing and update the Data Availability statement accordingly, including the ethical/legal restrictions, who imposed them, and contact information for a data access committee, ethics committee, or institutional body. | We agreed with this comment and revised the Data Availability Statement to make the restrictions clearer and more specific. The revised statement clarified that the data consisted of qualitative post-valuation interview and debrief transcripts, which could not be publicly shared as full transcripts, even after removal of direct identifiers, because they contained potentially identifying contextual information and participants did not consent to unrestricted public sharing of full transcripts.  We stated that these restrictions arose from the ethical approval/requirements of the KEMRI Scientific and Ethics Review Committee and institutional data governance requirements at the KEMRI-Wellcome Trust Research Programme. We retained the statement that relevant excerpts supporting the findings were included in the manuscript and that requests for de-identified excerpts could be directed to the KEMRI-Wellcome Trust Data Governance Committee: [DataGovernancecommittee@kemri-wellcome.org](mailto:DataGovernancecommittee@kemri-wellcome.org) |
| 3. | Please ensure that the Ethics Statement is available in its entirety at the beginning of the Methods section under a subheading ‘Ethics Statement’. | We agreed with this requirement. We revised the subheading from “Ethics approval statement” to “Ethics Statement” and moved the ethics statement in its entirety to the beginning of the Methods section, before the description of the study design, setting, and participants. |

**Table 2: Reviewer #1 comments**

|  | **Comment** | **Draft response/ action** |
| --- | --- | --- |
| 1. | The introduction could benefit from a brief description of what a valuation interview actually involves (cTTO and DCE tasks). Please consider adding 2–3 sentences describing the cTTO and DCE valuation tasks in accessible language for non-health-economics readers. | Thank you for this helpful comment. Although the cTTO and DCE tasks were briefly described in the Methods section in the original manuscript, we recognised that this explanation needed to appear earlier to orient non-health-economics readers. We therefore added a short, accessible description of the EQ-5D-5L valuation interview in the Introduction (Page 4), explaining that it involves two main tasks: composite time trade-off (cTTO) and discrete choice experiment (DCE), and briefly describing what each task asks participants to do.  To avoid repetition, we also revised the Methods section by shortening the description of cTTO and DCE and referring readers back to the earlier explanation. The Methods section now focuses on how participants were encouraged to think aloud during the valuation tasks and how these verbal reports informed the qualitative component of the study. |
| 2. | There is no mention of the growing body of EQ-5D-5L valuation work in sub-Saharan Africa (Ghana, Uganda, Ethiopia). Situating the work within this regional trend would strengthen the rationale. | We revised the Introduction (Page 5) to situate the Kenyan EQ-5D-5L national valuation study within the growing body of EQ-5D-5L valuation work in sub-Saharan Africa. Specifically, we added a paragraph noting that published EQ-5D-5L value sets are now available from Ethiopia, Uganda, Ghana, and Nigeria, reflecting increasing regional efforts to generate contextually relevant utility values for economic evaluation and health technology assessment. We then linked this regional trend to the Kenyan policy context by explaining that the need for locally derived utility values has become particularly salient as economic evaluation is increasingly being institutionalised in Kenya, including through the establishment of the Benefits Package and Tariffs Advisory Panel. This revision strengthened the rationale for the study by positioning it within both regional methodological developments and national priority-setting reforms. |
| 3. | Under methodology, please add a statement explaining the choice of SRQR over COREQ, or supplement with COREQ reporting. If COREQ was not followed, explain why SRQR was deemed more appropriate. | We revised the Methods section (Page 6) to clarify that SRQR was selected because it provides broader reporting guidance across qualitative study designs and was appropriate for this embedded qualitative study. We did not supplement with COREQ because SRQR and COREQ overlap substantially in their purpose and key reporting domains, and SRQR sufficiently covered the reporting requirements for this study design. |
| 4. | Inter-rater reliability or coding agreement is not described. Please describe the disagreement resolution process for coding, including whether a formal inter-rater reliability assessment was conducted | We revised the Methods section (Page 11) to describe the process for resolving differences in interpretation during coding and theme development. Specifically, we clarified that AK and JK reviewed the emerging codes and themes, and that areas of ambiguity or disagreement were discussed until consensus was reached. We also added that more complex interpretive issues were discussed with the broader authorship group where necessary. Finally, we clarified that formal inter-rater reliability was not assessed because the analysis was interpretive and thematic, rather than designed to quantify coding agreement. |
| 5. | No information is provided on how many valuation interviewers were involved in the main study or on their training and characteristics. Please provide basic characteristics of the valuation interviewer team | We revised the Methods section (Page 9) to provide basic information on the valuation interviewer team. Specifically, we added that 13 valuation interviewers, selected from 15 undergraduate trainees with health-related backgrounds, administered the computer-assisted EQ-5D-5L valuation interviews. We also clarified that they completed five days of training covering the EQ-5D and its application, interview content and interviewer skills, field preparation, quality-control procedures and reporting, mock interviews, and supervised trial interviews. In addition, we noted that before data collection, interviewers conducted pilot interviews over three weeks and received weekly feedback based on quality-control reports and discussions with the EQ-VT support team and study leads. |
| 6. | The finding that anxiety/depression was given less weight is important but under-explored. Please expand the discussion of why anxiety/depression was deprioritized, connecting translation challenges more explicitly to dimension-weighting patterns | We revised the Discussion section (Page 29) to explain why anxiety/depression appeared to be given less weight. We clarified that this lower salience may have reflected both participants’ prioritisation of more visible or functionally limiting dimensions, such as pain/discomfort, mobility, and usual activities, and linguistic/semantic ambiguity around the anxiety/depression domain. We linked this to the Results by noting that the Kiswahili term “kushuka moyo” was not always recognised as depression and was sometimes interpreted literally as a heart problem, discouragement, or loss of hope. We therefore clarified that lower weighting may partly reflect how the domain was translated and understood, rather than mental health being unimportant. |
| 7. | Only one participant had a chronic illness, yet personal lived experience was a major theme. Please clarify how the predominantly healthy sample engaged with severe health states, focusing on personal vs family/community experience | We revised the Results section (Page 16) to clarify that the theme referred to illness experience, including both participants’ own illness experiences and those they had observed among family or community members. We also clarified that although only one participant reported having a chronic illness, participants drew on these personal or observed experiences when interpreting severe hypothetical health states. To avoid ambiguity, we renamed the factor from “personal lived experience” to “illness experience: personal or observed” in Table 3 and used consistent wording in the Abstract, Results, and Conclusion. |
| 8. | Female participants were underrepresented (39.1%). Please note the female underrepresentation and whether it reflects the valuation study sample or purposive selection. | We revised the Methods section (page 7) to clarify that the sex distribution of the embedded qualitative sample reflected the purposive sampling approach used for the qualitative component, rather than the sex distribution of the national valuation study sample, which was broadly gender-balanced. |
| 9. | The discussion of conceptual equivalence could engage more critically with the limitations of this framework. The identification of missing dimensions may challenge conceptual equivalence rather than support it. | We revised the Discussion (Page 28) to engage more critically with conceptual equivalence. Specifically, we clarified that the close alignment between participants’ descriptions of health and the EQ-5D-5L domains supports conceptual equivalence at a broad/domain level. However, we also emphasised that this support was partial rather than complete, because participants highlighted aspects of health outside the descriptive system, namely vision, speech, broader mental health, and social relationships. We therefore revised the text to clarify that recognising the relevance of the core domains does not necessarily mean that the EQ-5D-5L descriptive system fully captures locally meaningful understandings of health. |
| 10. | No mention is made of whether any utility values were excluded from the final value set based on qualitative findings about comprehension challenges. Please clarify whether qualitative findings led to exclusion of any valuation data from the final value set. | We clarified this point in the Discussion, page 30. We explained that the qualitative findings did not lead to additional post-hoc exclusion of completed valuation data from the final value set. Instead, the qualitative findings were used to interpret how participants understood the valuation tasks and what contextual factors may have shaped their responses. We also retained the Results text showing that, during fieldwork, interviews were discontinued and participants replaced where participants could not meaningfully complete the tasks despite additional support. |

**Table 3: Reviewer #1 comments**

|  | **Comment** | **Draft response/ action** |
| --- | --- | --- |
| 1. | The methodological approach is not clear. The approach needs to be clarified in a way that makes the results easy to understand, and objectives and results should be linked more clearly. | We revised the manuscript to clarify the methodological approach and strengthen the link between the study objective, methods, and Results. In the Introduction, we revised the study objective to state more clearly that the study explored experiences of administering the EQ-5D-5L in Kenya, including how community participants understood and engaged with the EQ-5D-5L domains and valuation tasks, the factors that shaped their health-state preferences, and the administration challenges and coping strategies that emerged during valuation interviews.  In the Methods, we clarified the embedded qualitative design by explaining that the study was embedded because it was integrated within the Kenyan EQ-5D-5L national valuation study to examine how valuation interviews were understood, experienced, and administered in practice. In the Results, we revised the opening paragraph to show how the five interconnected themes reflected the study’s objectives: alignment between participants’ conceptualisations of health and EQ-5D-5L domains; factors shaping health-state preferences; tool/task-related and participant-related administration challenges; and coping and mitigation strategies used during fieldwork. |
| 2. | The overall sample of 23 is relatively small. It is documented that there were 16 debriefs. Did this include all 23 respondents? Please clarify the actual number of respondents and how many times data collection was done. | We revised the Data collection section to make the units of data collection clearer. We clarified that the study included 23 community participants, each of whom completed one post-valuation in-depth interview after the computer-assisted EQ-5D-5L valuation interview. We also clarified that the 16 debrief sessions were held with the valuation field team during fieldwork, comprising 14 debriefs with valuation interviewers and 2 with field supervisors. To further clarify the fieldwork process, we added information on the number, selection, training, piloting, and feedback process for the 13 valuation interviewers, and distinguished their role from that of the field supervisors who conducted the post-valuation interviews and facilitated the debrief sessions. |
| 3. | The section on reflexivity and research team adds no value to the overall paper. Please reconsider its inclusion; some content can be reorganized and included elsewhere. | We agreed that this section could be made more focused and better integrated into the manuscript. We retained a concise reflexivity statement because the manuscript is reported using SRQR, and SRQR includes researcher characteristics and reflexivity as a reporting item. However, we revised the section to reduce descriptive detail and focus more clearly on information relevant to interpretation of the findings. Specifically, we clarified the research team’s relevant expertise and the dual role of some team members in supporting the wider valuation study while also analysing experiences of that process. We also explained how this was managed analytically, including treating the data not only as operational feedback but also as evidence of how health states and valuation tasks were understood, and discussing emerging interpretations with collaborators who were not directly involved in Kenyan field supervision. |

We hope that the revised manuscript and our responses adequately address the editor’s and reviewers’ comments. We appreciate the time and consideration given to our manuscript and look forward to your decision.

Sincerely,

Dennis Waithaka, PhD

On behalf of all co-authors

KEMRI–Wellcome Trust Research Programme
