# Supplementary material for: Experiences administering the EuroQol five-dimension five-level (EQ-5D-5L) for valuation of health-related quality of life estimates in Kenya: An embedded qualitative study
Source: PLOS Glob Public Health. 2026 Nov 4;6(11):e0007568. doi: 10.1371/journal.pgph.0007568 (PMC13734439; doi:10.1371/journal.pgph.0007568)
Supplement: Peer review history

# Peer review: Decision Letter 0

**DOI:** 10.1371/journal.pgph.0007568.r001

**Authors:** Ali Aahil Noorali

16 Aug 2026

PGPH-D-26-02009

Experiences administering the EQ-5D-5L for valuation of health-related quality of life estimates in Kenya: An embedded qualitative study

PLOS Global Public Health

Dear Dr. Waithaka,

Thank you for submitting your manuscript to PLOS Global Public Health. After careful consideration, we feel that it has merit but does not fully meet PLOS Global Public Health’s publication criteria as it currently stands. Therefore, we invite you to submit a revised version of the manuscript that addresses the points raised during the review process.

Please submit your revised manuscript by Oct 15 2026 11:59PM. If you will need more time than this to complete your revisions, please reply to this message or contact the journal office at globalpubhealth@plos.org. When you're ready to submit your revision, log on to https://www.editorialmanager.com/pgph/ and select the 'Submissions Needing Revision' folder to locate your manuscript file.

Please include the following items when submitting your revised manuscript:

- A letter that responds to each point raised by the editor and reviewer(s). You should upload this letter as a separate file labeled 'Response to Reviewers'.
- A marked-up copy of your manuscript that highlights changes made to the original version. You should upload this as a separate file labeled 'Revised Manuscript with Track Changes'.
- An unmarked version of your revised paper without tracked changes. You should upload this as a separate file labeled 'Manuscript'.

Guidelines for resubmitting your figure files are available below the reviewer comments at the end of this letter.

As the corresponding author, your ORCID iD is verified in the submission system and will appear in the published article. PLOS supports the use of ORCID, and we encourage all coauthors to register for an ORCID iD and use it as well. Please encourage your coauthors to verify their ORCID iD within the submission system before final acceptance, as unverified ORCID iDs will not appear in the published article. *Only*  the individual author can complete the verification step; PLOS staff *cannot*  verify ORCID iDs on behalf of authors.

We look forward to receiving your revised manuscript.

Kind regards,

Ali Aahil Noorali, MD, MPH, MBA

Guest Editor

PLOS Global Public Health

**Journal Requirements:**

1. Please amend your detailed Financial Disclosure statement. This is published with the article. It must therefore be completed in full sentences and contain the exact wording you wish to be published.

i. Please clarify all sources of financial support for your study. List the grants, grant numbers, and organizations that funded your study, including funding received from your institution. Please note that suppliers of material support, including research materials, should be recognized in the Acknowledgements section rather than in the Financial Disclosure.

ii. State the initials, alongside each funding source, of each author to receive each grant. For example: "This work was supported by the National Institutes of Health (####### to AM; ###### to CJ) and the National Science Foundation (###### to AM)."

iii. State what role the funders took in the study. If the funders had no role in your study, please state: “The funders had no role in study design, data collection and analysis, decision to publish, or preparation of the manuscript.”

iv. If any authors received a salary from any of your funders, please state which authors and which funders.

2. We note that you have indicated that there are restrictions to data sharing for this study. For studies involving human research participant data or other sensitive data, we encourage authors to share de-identified or anonymized data. However, when data cannot be publicly shared for ethical reasons, we allow authors to make their data sets available upon request. For information on unacceptable data access restrictions, please see http://journals.plos.org/globalpublichealth/s/data-availability#loc-unacceptable-data-access-restrictions.

Before we proceed with your manuscript, please address the following prompts:

a) If there are ethical or legal restrictions on sharing a de-identified data set, please explain them in detail (e.g., data contain potentially identifying or sensitive patient information, data are owned by a third-party organization, etc.) and who has imposed them (e.g., a Research Ethics Committee or Institutional Review Board, etc.). Please also provide contact information for a data access committee, ethics committee, or other institutional body to which data requests may be sent.

b) If there are no restrictions, please upload the minimal anonymized data set necessary to replicate your study findings to a stable, public repository and provide us with the relevant URLs, DOIs, or accession numbers. Please see http://www.bmj.com/content/340/bmj.c181.long for guidelines on how to de-identify and prepare clinical data for publication. For a list of recommended repositories, please see https://journals.plos.org/globalpublichealth/s/recommended-repositories. You also have the option of uploading the data as Supporting Information files, but we would recommend depositing data directly to a data repository if possible.

Please update your Data Availability statement in the submission form accordingly.

3. Please ensure that your Ethics Statement is available in its entirety at the beginning of your Methods section, under a subheading 'Ethics Statement'.

If the reviewer comments include a recommendation to cite specific previously published works, please review and evaluate these publications to determine whether they are relevant and should be cited. There is no requirement to cite these works unless the editor has indicated otherwise.

**Additional Editor Comments (if provided):**

[Note: HTML markup is below. Please do not edit.]

Reviewers' comments:

Reviewer’s Responses to Questions

**Comments to the Author**

1. Does this manuscript meet PLOS Global Public Health’s publication criteria?

Reviewer #1: Yes

Reviewer #2: Yes

**********

2. Has the statistical analysis been performed appropriately and rigorously?

Reviewer #1: N/A

Reviewer #2: Yes

**********

3. Have the authors made all data underlying the findings in their manuscript fully available (please refer to the Data Availability Statement at the start of the manuscript PDF file)?

The PLOS Data policy

Reviewer #1: Yes

Reviewer #2: Yes

**********

4. Is the manuscript presented in an intelligible fashion and written in standard English?

Reviewer #1: Yes

Reviewer #2: Yes

**********

**Reviewer #1:**  1. The introduction could benefit from a brief description of what a valuation interview actually involves (cTTO and DCE tasks). Readers from public health backgrounds may not be familiar with these methodological details. Please consider adding 2–3 sentences describing the cTTO and DCE valuation tasks in accessible language for non-health-economics readers.

2. While the Kenyan policy context is well-described, there is no mention of the growing body of EQ-5D-5L valuation work in sub-Saharan Africa (Ghana, Uganda, Ethiopia) that this study joins, situating the work within this regional trend would strengthen the rationale. Please consider adding a sentence noting the growing body of EQ-5D-5L valuation work in sub-Saharan Africa to situate this study within a regional trend. [https://www.sciencedirect.com/science/article/pii/S2212109924000785; https://pmc.ncbi.nlm.nih.gov/articles/PMC8627844/].

3. Under methodology, please add a statement explaining the choice of SRQR over COREQ, or supplement with COREQ reporting. If COREQ was not followed, explain why SRQR was deemed more appropriate.

4. Inter-rater reliability or coding agreement is not described. The manuscript states that AK and JK "reviewed the emerging analysis and discussed areas of ambiguity or disagreement" (lines 165–167), but does not describe the process for resolving disagreements or whether a formal inter-rater reliability assessment was conducted. Please describe the disagreement resolution process for coding (e.g., consensus through discussion, third-party arbitration).

5. No information is provided on how many valuation interviewers were involved in the main study or on their training and characteristics. This information would help readers assess whether the reported challenges reflect systematic issues or interviewer-specific variation. Please provide basic characteristics of the valuation interviewer team (number, training background).

6. Results: The finding that anxiety/depression was given less weight (lines 217–220) is important but under-explored. Why was this dimension deprioritized? Was it due to stigma around mental health, different conceptualizations of anxiety/depression, or the Kiswahili translation of "kushuka moyo"? The authors raise the translation issue but do not fully connect it to the dimension-weighting finding. Please expand the discussion of why anxiety/depression was deprioritized, connecting translation challenges more explicitly to dimension-weighting patterns.

7. Only one participant had a chronic illness (4.3%), yet personal lived experience was a major theme. This raises a question: how did the predominantly healthy sample engage with severe health states? Did they draw on family/community experience rather than personal experience? This distinction could be clarified. Please consider clarifying how the predominantly healthy sample engaged with severe health states focusing on personal vs. family/community experience.

8. Female participants were underrepresented (39.1%). While the purposive sampling sought variation, the reasons for this gender distribution and whether it reflects the valuation study’s overall sample composition should be noted. Please note the female underrepresentation and whether it reflects the valuation study sample or purposive selection.

9. The discussion of "conceptual equivalence" (lines 453–460) could engage more critically with the limitations of this framework. While participants' descriptions aligned with EQ-5D-5L domains, the identification of missing dimensions (vision, speech, broader mental health, social relationships) arguably challenges conceptual equivalence rather than supporting it. The authors note this but could more explicitly discuss whether the absence of these dimensions represents a meaningful limitation of the EQ-5D-5L for Kenyan populations. Please consider engaging more critically with the "conceptual equivalence" framework, does the identification of missing dimensions challenge or support equivalence claims?

10. No mention is made of whether any utility values were excluded from the final value set based on qualitative findings about comprehension challenges. If so, this should be reported. If not, the implications for value set validity should be discussed. Please clarify whether qualitative findings led to exclusion of any valuation data from the final value set.

**Reviewer #2:**  The paper discusses an interesting concept. However there are a few issue that need to be addressed.

1. The methodological approach is not clear. The approach needs to be clarified in a way that makes the result easy to understand. In addition to this its important to link both the objectives and the results. What are the key objectives, what is the main focus of the paper. From the paper it does not come out clear what is supposed to be achieved by the results.

2. The overall sample of 23 is relatively small. However, it is documented that there were 16 debriefs did this include all the 23 respondents. If this is the case then clarify how many times data collection was done and if the tool was collected from 23 people 16 times then the sample should be higher than 23 since each entry is counted as one. Otherwise clarify the actual number of respondents involved in the study from what we have its unclear as embedded qualitative studies have elements of both quantitative and qualitative data collection.

3. The inclusion of the section on reflexivity and research team adds no value to the overall paper. Its a description of the teams coordination during the study. Please reconsider its inclusion some section of this can be reorganized and included in other sections of the paper where its more relevant.

**********

what does this mean?). If published, this will include your full peer review and any attached files.

**Do you want your identity to be public for this peer review?**  If you choose “no”, your identity will remain anonymous but your review may still be made public.

For information about this choice, including consent withdrawal, please see our Privacy Policy

Reviewer #1: No

Reviewer #2: No

**********

[NOTE: If reviewer comments were submitted as an attachment file, they will be attached to this email and accessible via the submission site. Please log into your account, locate the manuscript record, and check for the action link "View Attachments". If this link does not appear, there are no attachment files.]

**Figure Resubmissions:**

# Author comment: Author response to Decision Letter 1

**DOI:** 10.1371/journal.pgph.0007568.r002

31 Aug 2026

Attachment Submitted filename: Response to Reviewers.docx

# Peer review: Decision Letter 1

**DOI:** 10.1371/journal.pgph.0007568.r003

**Authors:** Julia Lohmann

19 Oct 2026

Experiences administering the EuroQol five-dimension five-level (EQ-5D-5L) for valuation of health-related quality of life estimates in Kenya: An embedded qualitative study

PGPH-D-26-02009R1

Dear Dr. Waithaka,

We are pleased to inform you that your manuscript 'Experiences administering the EuroQol five-dimension five-level (EQ-5D-5L) for valuation of health-related quality of life estimates in Kenya: An embedded qualitative study' has been provisionally accepted for publication in PLOS Global Public Health.

Before your manuscript can be formally accepted you will need to complete some formatting changes, which you will receive in a follow up email. A member of our team will be in touch with a set of requests.

Please note that your manuscript will not be scheduled for publication until you have made the required changes, so a swift response is appreciated.

IMPORTANT: The editorial review process is now complete. PLOS will only permit corrections to spelling, formatting or significant scientific errors from this point onwards. Requests for major changes, or any which affect the scientific understanding of your work, will cause delays to the publication date of your manuscript.

If your institution or institutions have a press office, please notify them about your upcoming paper to help maximize its impact. If they'll be preparing press materials, please inform our press team as soon as possible -- no later than 48 hours after receiving the formal acceptance. Your manuscript will remain under strict press embargo until 2 pm Eastern Time on the date of publication. For more information, please contact globalpubhealth@plos.org.

Thank you again for supporting Open Access publishing; we are looking forward to publishing your work in PLOS Global Public Health.

Best regards,

Julia Lohmann

Academic Editor

PLOS Global Public Health

***********************************************************

Reviewer Comments (if any, and for reference):

Reviewer’s Responses to Questions

**Comments to the Author**

Reviewer #1: All comments have been addressed

Reviewer #2: All comments have been addressed

**********

publication criteria?

Reviewer #1: Yes

Reviewer #2: Yes

**********

3. Has the statistical analysis been performed appropriately and rigorously?

Reviewer #1: Yes

Reviewer #2: Yes

**********

4. Have the authors made all data underlying the findings in their manuscript fully available (please refer to the Data Availability Statement at the start of the manuscript PDF file)?

The PLOS Data policy

Reviewer #1: Yes

Reviewer #2: Yes

**********

5. Is the manuscript presented in an intelligible fashion and written in standard English?

Reviewer #1: Yes

Reviewer #2: Yes

**********

Reviewer #1: Congratulations to the researchers.

Reviewer #2: I have no further comments all my issues have been addressed

**********

what does this mean?). If published, this will include your full peer review and any attached files.

**Do you want your identity to be public for this peer review?**  If you choose “no”, your identity will remain anonymous but your review may still be made public.

For information about this choice, including consent withdrawal, please see our Privacy Policy

Reviewer #1: No

Reviewer #2: No

**********
